# Race/Ethnicity, Socioeconomic Status, and Polypharmacy among Older Americans

**DOI:** 10.3390/pharmacy7020041

**Published:** 2019-04-25

**Authors:** Shervin Assari, Mohsen Bazargan

**Affiliations:** 1 Department of Family Medicine, Charles R. Drew University of Medicine and Science, Los Angeles, CA 90059, USA; mohsenbazargan@cdrewu.edu; 2 Department of Family Medicine, University of California Los Angeles (UCLA), Los Angeles, CA 90095, USA

**Keywords:** social determinants, socioeconomic status, polypharmacy, ethnicity, race, African Americans, Blacks

## Abstract

**Background:** Very few studies with nationally representative samples have investigated the combined effects of race/ethnicity and socioeconomic position (SEP) on polypharmacy (PP) among older Americans. For instance, we do not know if prevalence of PP differs between African Americans (AA) and white older adults, whether this difference is due to a racial gap in SEP, or whether racial and ethnic differences exist in the effects of SEP indicators on PP. **Aims:** We investigated joint effects of race/ethnicity and SEP on PP in a national household sample of American older adults. **Methods:** The first wave of the University of Michigan National Poll on Healthy Aging included a total of 906 older adults who were 65 years or older (80 AA and 826 white). Race/ethnicity, SEP (income, education attainment, marital status, and employment), age, gender, and PP (using 5+ medications) were measured. Logistic regression was applied for data analysis. **Results:** Race/ethnicity, age, marital status, and employment did not correlate with PP; however, female gender, low education attainment, and low income were associated with higher odds of PP among participants. Race/ethnicity interacted with low income on odds of PP, suggesting that low income might be more strongly associated with PP in AA than white older adults. **Conclusions:** While SEP indicators influence the risk of PP, such effects may not be identical across diverse racial and ethnic groups. That is, race/ethnicity and SEP have combined/interdependent rather than separate/independent effects on PP. Low-income AA older adults particularly need to be evaluated for PP. Given that race and SEP have intertwined effects on PP, racially and ethnically tailored interventions that address PP among low-income AA older adults may be superior to universal interventions and programs that ignore the specific needs of diverse populations. The results are preliminary and require replication in larger sample sizes, with PP measured directly without relying on individuals’ self-reports, and with joint data collected on chronic disease.

## 1. Introduction

Polypharmacy (PP), defined as the concomitant use of several prescribed medicines, increases the risk of inappropriate use of medications as well as undesired drug interactions [1,2,3]. Although it is differently defined across various studies [4], PP is commonly found to be correlated with a variety of health threats including drug-drug interactions and reduced adherence to necessary medications [5,6]. As PP is linked to cognitive decline [5,6], low quality of life [7], depression [8], function [9], psychological distress [10], and unintentional falls [5,6], prevention of PP may considerably reduce the costs to patients, families, the healthcare system, and society [11]. As PP increases the risk of morbidity and mortality of older adults [12], it is essential to conduct epidemiological studies, particularly on nationally representative samples, that have the potential to inform programs and interventions that can be used to reduce the burden of PP on society as a whole [13].

For several reasons, epidemiologists, economists, and clinicians are showing increasing interest in understanding the social patterning of PP in older adult populations. First, PP is a costly phenomenon [11]. In addition, PP is linked to undesired health outcomes such as hospitalization [14]. PP should be considered a serious health risk, particularly for older adults, as it increases adverse drug reactions by 75% in this age group [15,16]. Drug interactions that are a consequence of inappropriate PP are responsible for some of the hospitalizations among older adults [17]. Almost half of such hospitalizations can be avoided by the prevention of these inappropriate drug interactions [17]. In addition, PP is linked to mortality [14,18]. Finally, the problem of PP especially becomes a common problem as the population ages [19].

It has been shown that PP is particularly common in older adults [20,21,22,23] as they receive multiple prescribed medications [21] for their diagnosed chronic medical [1,24,25,26] and psychiatric [27] conditions. PP is particularly common in older adults who have multi-morbidities and comorbidities [22,23]. In addition to experiencing a higher prevalence of PP, older people are also more vulnerable to its adverse effects due to their frailty status, multimorbidity, and impaired cognitive capacity [28]. Moreover, age-related physiological changes make older adults more vulnerable to medical complications of PP compared to younger individuals [28]. In many countries, as the average age of populations increases, PP is becoming more common [29]. Thus, there is a need for more studies that help us understand the risk factors of PP.

The occurrence of PP in society, however, is not random but rather follows a social gradient [30]. PP is particularly common in racial and ethnic minority and low socioeconomic position (SEP) individuals who have multiple chronic conditions [31]. In addition to a high prevalence of PP [32], these populations are vulnerable to low health literacy and cognitive dysfunction [33,34]. These combined factors make individuals in these groups particularly prone to the adverse effects of PP [28].

Race and ethnicity may be among the key determinants of PP [35]. Across various race/ethnic groups, PP may be particularly common in African-American (AA) individuals [36] who have a disproportionately high risk of chronic disease [31]. AA patients are less likely to receive new, most effective, or simplified medication combinations [37,38,39,40,41]. As a result of poor access to such combined/simple medication regimens, AA patients are more likely to be prescribed with regimens composed of multiple, older, complex, and generic medications [4,36]. The result is an increased risk of PP, inappropriate medication use, and medication non-adherence in the AA community [42]. From the pool of epidemiological studies that have focused on PP [43,44,45], however, very few compare white and AA people [4,36]. We are not aware of any previous studies with a nationally representative sample investigating the combined effects of race/ethnicity and SEP on PP.

In addition to race/ethnicity, SEP indicators also seem to impact the risk of PP. Low education attainment and unemployment [46] are shown to impact the prevalence and burden of PP by directly reducing disease burden [29] or through other mechanisms [34]. Education and other SEP indicators seem to also alter population access to healthcare, which may increase the risk of PP [47].

Existing epidemiological information is very limited regarding the combined effects of race/ethnicity and SEP on PP [36,48,49]. From this limited number of existing studies, most studies have recruited local samples, leaving a knowledge gap regarding nationally representative estimates [36]. Bazargan et al. have shown that 75% of underserved older AA adults in South Los Angeles (n = 400) had PP (were taking 5+ medications per day) [36]. The same study went beyond studying polypharmacy and documented a 70% risk of inappropriate drug use. In that study, while gender, the number of providers, potentially inappropriate medication use, and multimorbidity were risk factors for PP, no SEP factors were found to affect PP in AA older adults [36]. There is an ongoing need to understand how race and SEP influence epidemiology of PP in American older adults.

Epidemiological studies that investigate the risk of PP by race/ethnicity and SEP may introduce new insights regarding individuals who are at an increased risk of potentially inappropriate medication use [50,51,52]. Such knowledge is essential for national strategies that aim to eliminate racial disparities in PP in the US. The findings derived from such epidemiological studies may be one step towards addressing the unmet health needs of AAs. Such information may be used to design, implement, and evaluate evidence-based programs that specifically target high-risk AA people who are at risk of potentially inappropriate medication use. Unfortunately, among various aspects of the health of AA people, PP is an understudied area [4,36,48,49].

### Aims

This study recruited a nationally representative sample of older adults to investigate how race/ethnicity and SEP impact the risk of PP (taking five or more medications) among American older adults.

## 2. Methods

### 2.1. Design and Setting

This study used data from the first wave of the National Poll on Healthy Aging (NPHA, 2017). The NPHA is an online survey of older adults in the United States which uses a nationally representative sample. Conducted by the University of Michigan (UM) Institute for Healthcare Policy and Innovation (IHPI), the main aim of the UM-NPHA is to monitor changes in the health and well-being of older Americans.

### 2.2. National Poll on Healthy Aging 2017

The NPHA uses the Knowledge Networks (GFK KnowledgePanel) framework for sampling. This is an online internet panel which is nationally representative of US adults. The NPHA gathers data on the health and wellbeing of American adults who are at least 50 years old. Using random sampling, the NPHA provides an opportunity to study the combined effects of race/ethnicity, gender, and SEP on the daily life of older adults in the US. The study collects data on demographics, SEP indicators, social networks, and healthcare use. The study website is available here: https://www.healthyagingpoll.org/.

### 2.3. Analytical Sample

The current study included 906 older adults who were 65 years or older (comprising 80 AA and 826 non-Hispanic white individuals). Exclusion criteria for this study were (1) having an age lower than 65 years, (2) being of a racial and ethnic background other than AA/Black or white, and (3) being Hispanic [53].

### 2.4. Ethics

The University of Michigan Institutional Review Board (IRB) exempted the review of the NPHA study protocol. All NPHA participants signed informed consent.

### 2.5. Study Measures

Study variables included race/ethnicity, gender, age, marital status, employment, and physical SRH.

#### 2.5.1. Independent Variables

*Race/Ethnicity.* Self-identified race/ethnicity was the moderator. Ethnicity was a dichotomous variable (non-Hispanic white 0 (the reference group), AA 1).

*Educational Attainment.* Education in this study was measured as an interval variable ranging from 1 to 12. A higher score was indicative of higher education attainment.

*Employment Status.* Employment status was operationalized as a dichotomous variable (unemployed 0 versus employed 1).

*Marital Status.* Marital status was a dichotomous variable (not married 0 versus married 1).

*Poverty Status.* Poverty status was calculated as a 21-level interval variable. These levels were based on household income which was calculated as an interval variable ranging from 1 to 21. Some examples of the income levels are 9 ($30,000 to $34,999), 12 ($50,000 to $59,999), 15 ($85,000 to $99,999), and 17 ($125,000 to $149,999). A higher poverty status score was indicative of lower SEP (less household income).

#### 2.5.2. Dependent Variable

Polypharmacy was measured using self-reporting of the number of prescribed medicines. The exact question used was “How many different prescription medications are you currently taking?” Taking 5 or more medicines was considered as PP. This variable was treated as a dichotomous variable (no 0, yes 1) [1]. No additional instructions were given to the participants about prescription medications (please see our discussion of limitations).

#### 2.5.3. Covariates

*Demographic Variables.* Gender and age were the study covariates. Age was treated as an interval variable, measured in years. Gender was treated as a dichotomous measure (male 0, female 1).

### 2.6. Statistical Analysis

Data analysis was performed using Stata 15.00 (Stata Corp., College Station, TX, USA). To describe the sample overall and by race/ethnicity, we reported the frequency (%) and mean (SD). We used chi-square as well as independent samples t-tests to compare AA and white older adults for the study variables. We used six logistic regression models. Model 1 only included the main effect of race/ethnicity. Model 2 included the main effects of the SEP indicators. Model 3 included race/ethnicity as well as the SEP indicators. Model 4 included two-by-two interaction terms between race/ethnicity and the SEP indicators. Model 5 and Model 6 tested the effects of SEP indicators for non-Hispanic white and AA older adults, respectively. Regression coefficients, standard errors (SE), 95% CI values, and *p*-values were reported.

## 3. Results

### 3.1. Descriptive Statistics

Table 1 describes demographic factors, SEP indicators, and PP overall and by race/ethnicity. The sample included 906 older adults who were 65 years of age or older (80 AA and 826 white). About 19.5% and 19.0% of white and AA older adults reported PP, respectively. AA older adults were less likely to be married, reported lower income, and had lower educational attainment (*p* < 0.05). AA and white older adults did not differ in employment status (*p* < 0.05).

### 3.2. Separate and Combined Effects of Race/Ethnicity and SEP on PP in the Pooled Sample

Table 2 shows a summary of the results of four logistic regressions with PP as the outcome in the pooled sample. Based on Model 1, which only included race/ethnicity, gender, and age, race/ethnicity was not associated with PP (*p* = 0.967). Based on Model 2, which only included SEP indicators, education (*p* = 0.004), and employment (*p* = 0.002), but not poverty status (*p* = 0.980), were associated with PP. Based on Model 3, which included race/ethnicity and SEP indicators, education (*p* = 0.037) and employment (*p* = 0.006), but not race/ethnicity (*p* = 0.643) or poverty status (*p* = 0.714), were associated with PP. Based on Model 4, which also showed 2-by-2 interaction terms between SEP and race/ethnicity, poverty status showed an interaction with race/ethnicity (*p* = 0.041), with poverty status having a stronger effect on the prevalence of PP for AA than non-Hispanic white older adults (Table 2).

### 3.3. SEP Determinants of Polypharmacy in White and AA Older Adults

Table 3 shows the results of two logistic regressions with PP as the outcome in non-Hispanic white and AA older adults. In non-Hispanic whites, income was not correlated with PP (*p* = 0.863). In AA older adults, however, low income was associated with PP (*p* = 0.031) (Table 3).

## 4. Discussion

Using a national sample of American older adults, this study has investigated the joint effects of race/ethnicity and SEP on social patterning of PP in the US. We found that SEP but not race/ethnicity impacts PP and that the effects of SEP indicators depend on race/ethnicity. That is, low income has a stronger effect as a risk factor for PP for AA than white older adults in the US.

We found a lower risk of PP for females than males, which is different from what has been concluded in most of the previous literature [54,55]. Although not all [56], at least some [54,55,57] studies have documented a higher prevalence of PP for females than males. Beyond PP, inappropriate drug use is also more common in women [54,55,58], an association that seems to exist above and beyond age, SEP, and health [55]. This apparent contradiction may be due to better adherence of women to prescriptions [58]. The same gender differences are shown in low SEP older adults [59]. While men may be at a higher risk of cardiac PP, women are at an increased risk of non-cardiac PP [29]. Compared to men, women report more non-fatal chronic diseases [60,61] and are more likely to seek care and take medications for such conditions [62,63,64]. Women recognize [65] and communicate [66] their symptoms to their physicians and healthcare providers better than men. These gender differences are in part due to gender roles and gender socialization [67,68,69]. Women are more likely to adopt the sick role, which is socially learned [70,71]. Our finding, however, contradicts past research on higher PP in female as opposed to male older Americans.

Within older adults, we did not find any effect of age on PP. The effects of age on PP are well-established. At least some of the effects of age on PP are due to chronic diseases. In our study, however, we did not have information on chronic diseases. As individuals age, chronic diseases accumulate over time; such a growing catalogue of conditions means more pharmacological treatments prescribed for the individual [24]. As people reach their early sixties, the majority of individuals have at least two diagnosed chronic disorders that require treatment [25], and as individuals reach their late sixties, more than 25% of those older adults have three or more diagnosed chronic diseases that require treatment [26]. The effect of age on PP may also be different across racial groups, as chronic disease develops several years earlier in AA people than whites [72], suggesting that screening for PP should probably start earlier for low SEP AA people.

Several SEP indicators were found in this study that were correlated with PP. Low education attainment and unemployment were risk factors for PP. In the Swedish Panel Study of Living Conditions of the Oldest Old (SWEOLD 2002), which included a nationally representative sample of Swedish older adults (age ≥ 77 years), people with lower education were more likely to have PP. However, this association was fully explained by physical health [46]. In another study, high risks of PP in people with low education could not be explained by disease burdens [29]. In a recent study among AA people, however, education was positively linked to PP. The authors suggested that in the AA population, education may enable healthcare access, so AA people with higher education can get their conditions diagnosed and treated more frequently [47].

With PP becoming widespread due to the aging of the population [73], there is an increasing need to understand the social patterning of PP by conducting epidemiological studies and to conduct programs that can minimize the SEP and racial disparities in PP and inappropriate drug use. The results reported here may contribute to designing the interventions that may reduce PP in AA and low SEP people [36,74,75]. Current strategies are inefficient in reducing PP [76], emphasizing the necessity of conducting interventions that are most effective in reducing inappropriate PP for low SEP and minority people [36,76].

Although race/ethnicity and income were not shown to have major effects on PP, low income AA people were at an increased risk of PP. This is not the first study documenting an interaction between SEP and race/ethnicity on health outcomes [77,78]. However, this is probably the first study focusing on PP as the outcome. Differential health effects of education [79], income [80,81], employment [82], and marital status [83] are shown between whites and AA people. Such differential effects (SEP showing different effects by race) are very distinct from differential exposures (SEP is partially why racial minorities have worse health); however, the two mechanisms are complementary, as both contribute to existing health disparities [77]. These patterns emerge because populations with very equal SEP have unequal health needs, purchasing power, life conditions, environment, and healthcare access [77,78].

### 4.1. Implications

The findings reported here may be implemented by informing clinical practice as well as design and implement of public health programs and interventions that can ultimately eliminate or at least reduce racial and economic disparities in PP. Although the ultimate goal goes beyond reducing PP and should target inappropriate drug use, given the association between the two, knowledge of social epidemiology of PP is still relevant to reducing such disparities. We suggest that a comprehensive evaluation of PP and inappropriate drug use is needed, particularly in low SEP and AA older individuals.

Although in the discussion of this paper we spoke about the importance of preventing inappropriate PP, our outcome variable was simply PP. Some of our sample may be appropriate and some may be inappropriate PP. There is a need to conduct future research on inappropriate PP by race and SES. It should also be noted that in this study we only made conclusions based on PP rather than inappropriate PP. As mentioned above, we only know who is poly-medicated, regardless of knowing whether that is inappropriate PP.

Our results should be regarded as preliminary because we used a self-reported number of medicines given by the patient to measure polypharmacy. This is a major issue to consider, as the information that aging people can provide including that regarding medication may not always be reliable. Older adults may confuse medicines and doses and may even consider products such as vitamins and over the counter products as medications. Moreover, we were not able to gather information about possible cognitive problems in our patients. Unmeasured poor cognitive performance may have been a major source of bias in this study.

### 4.2. Limitations

The current study had several limitations. First, with a cross-sectional design, causal associations were not plausible. More research on the social determinants of PP is needed with a longitudinal design. Second, PP (our dependent variable) was defined based on a self-reported number of prescribed medications. This approach is prone to recall bias [84]. Future research should collect medication data from pharmacy charts or insurance claims, rather than simply relying on patients’ self-reports. Third, many relevant pieces of information such as health status and chronic disease were not included. Other than chronic disease [85], access to healthcare systems, health care coverage, regular places of care [86] and the number of healthcare providers [36] impacts PP. Unfortunately, this study did not include any data on chronic diseases which are influenced by SEP, race/ethnicity, and their intersections. Future research should include types of medical and psychiatric conditions as well as types of prescribed medications. Available data on chronic disease provides us the opportunity to define inappropriate use of medications using various algorithms including but not limited to STOPP/START criteria [87,88,89]. In the absence of data on chronic disease, we have only referred to our outcome as PP instead of inappropriate PP or inappropriate use of medications. Such data will help detect inappropriate medication use. All the variables in this study were limited to the individual level with no data collected from providers. Future research should investigate both patient and provider factors. Finally, this study had a very low sample of AA participants. Thus, there is a need for replication of this study in future, particularly with a larger number of AA subjects, longitudinal design, and a more comprehensive measure of PP and confounders. Despite these limitations, this study is among the first to report on the combined effects of race/ethnicity and SEP on PP, and how white and AA older adults differ in PP risk factors.

## 5. Conclusions

To conclude, race/ethnicity and SEP have joint (non-linear) effects on PP within the general population of older Americans. To be more specific, low income seems to have a stronger effect on PP for AA than white older Americans. There is a need to test culturally acceptable programs and interventions that consider specific needs due to intersections of race, ethnicity, and SEP. Such programs may be superior to universal programs that target PP.

## Figures and Tables

**Table 1:** Descriptive results for the overall sample (n = 906).

	All (n = 906)	Whites (n = 826)	AA (n = 80)
	Mean (Standard Error (SE))	Mean (SE)	Mean (SE)
Age	71.07 (0.14)	71.15 (0.15)	70.29 (0.46)
Poverty *	9.35 (0.14)	9.25 (0.15)	10.43 (0.45)
Education (1)–(4) *	2.92 (0.03)	2.96 (0.03)	2.54 (0.10)
	**n (%)**	**n (%)**	**n (%)**
Gender			
Male	453 (48.92)	414 (48.94)	39 (48.75)
Female	473 (51.08)	432 (51.06)	41 (51.25)
Employed			
No	778 (84.20)	709 (83.91)	69 (87.34)
Yes	146 (15.80)	136 (16.09)	10 (12.66)
Married *			
No	295 (31.86)	252 (29.79)	43 (53.75)
Yes	631 (68.14)	594 (70.21)	37 (46.25)
Polypharmacy *			
No	744 (80.52)	680 (80.47)	64 (81.01)
Yes	180 (19.48)	165 (19.53)	15 (18.99)

Source: The National Poll on Healthy Aging 2017. AA, African Americans * *p* < 0.05.

**Table 2:** Four logistic regression models with polypharmacy as the outcome in the overall sample.

	OR(SE)	95% OR	Z	*p*
**Model 1 (All; n = 906) Race Model**				
Race/ethnicity (AA)	0.99 (0.30)	0.55–1.78	−0.04	0.967
Gender (female)	0.75 (0.13)	0.54–1.05	−1.69	0.090
Age	1.02 (0.02)	0.98–1.06	1.10	0.269
**Model 2 (All; n = 906) SEP Model**				
Gender (female)	0.69 (0.11)	0.50–0.96	−2.24	0.025
Age	1.01 (0.02)	0.98–1.05	0.78	0.434
Marital status	0.99 (0.18)	0.69–1.42	−0.05	0.959
Education	0.76 (0.07)	0.63–0.92	−2.87	0.004
Employment	0.42 (0.12)	0.24–0.73	−3.07	0.002
Poverty	1.00 (0.02)	0.96–1.04	−0.02	0.980
**Model 3 (All; n = 906) Race and SEP Model**				
Race (AA)	0.87 (0.27)	0.47–1.59	−0.46	0.643
Gender (female)	0.68 (0.12)	0.49–0.96	−2.17	0.030
Age	1.01 (0.02)	0.97–1.05	0.65	0.515
Marital status	1.02 (0.20)	0.69–1.51	0.11	0.913
Education	0.80 (0.08)	0.65–0.99	−2.08	0.037
Employment	0.45 (0.13)	0.25–0.79	−2.77	0.006
Poverty	1.01 (0.02)	0.96–1.06	0.37	0.714
**Model 4 (All; n = 906) Race by SEP Model**				
Race (AA)	0.06 (0.11)	0.00–2.28	−1.52	0.129
Gender (female)	0.66 (0.12)	0.47–0.94	−2.34	0.019
Age	1.02 (0.02)	0.98–1.06	0.86	0.390
Marital status	1.11 (0.24)	0.74–1.69	0.51	0.609
Education	0.78 (0.09)	0.63–0.96	−2.29	0.022
Employment	0.44 (0.13)	0.24–0.80	−2.72	0.007
Poverty	1.00 (0.02)	0.95–1.04	−0.18	0.858
Education × Race (AA)	1.37 (0.51)	0.66–2.86	0.84	0.401
Poverty × Race (AA)	1.22 (0.12)	1.01–1.48	2.04	0.041
Employed × Race (AA)	1.77 (2.10)	0.17–18.11	0.48	0.632
Married × Race (AA)	0.38 (0.27)	0.10–1.50	−1.38	0.167

Source: The National Poll on Healthy Aging 2017. Legend: AA, African Americans; SEP, socioeconomic position; OR, odds ratio.

**Table 3:** Results of two logistic regressions with polypharmacy as the outcome in race/ethnic groups.

	OR(SE)	95% OR	Z	*p*
**Model 3 (White; n = 826)**				
Gender (female)	0.69 (0.13)	0.48–0.99	−2.04	0.042
Age	1.02 (0.02)	0.97–1.06	0.75	0.453
Marital status	1.12 (0.24)	0.74–1.70	0.54	0.587
Education	0.78 (0.09)	0.63–0.97	−2.25	0.024
Employment	0.44 (0.13)	0.24–0.80	−2.72	0.007
Poverty	1.00 (0.02)	0.95–1.05	−0.17	0.863
**Model 3 (AA; n = 80)**				
Gender (female)	0.34 (0.25)	0.08–1.42	−1.48	0.139
Age	1.06 (0.09)	0.91–1.25	0.77	0.441
Marital status	0.33 (0.24)	0.08–1.39	−1.51	0.131
Education	1.08 (0.40)	0.52–2.21	0.20	0.840
Employment	1.00 (1.20)	0.10–10.49	0.00	0.999
Poverty	1.26 (0.13)	1.02–1.54	2.16	0.031

Source: The National Poll on Healthy Aging 2017. AA, African Americans.
